# Overview of Nanoparticle-Based Approaches for the Combination of Photodynamic Therapy (PDT) and Chemotherapy at the Preclinical Stage

**DOI:** 10.3390/cancers14184462

**Published:** 2022-09-14

**Authors:** Luca Menilli, Celeste Milani, Elena Reddi, Francesca Moret

**Affiliations:** 1 Department of Biology, University of Padova, 35100 Padova, Italy; 2 Institute of Organic Synthesis and Photoreactivity, ISOF-CNR, 40129 Bologna, Italy

**Keywords:** photodynamic therapy, chemotherapy, photosensitizers, nanoparticles, upconverting nanoparticles, synergistic combination, tumor microenvironment responsive drugs, biomimetic nanoparticles, targeted tumor therapy

## Abstract

**Simple Summary:**

The present review represents the outstanding and promising recent literature reports (2017–2022) on nanoparticle-based formulations developed for anticancer therapy with photodynamic therapy (PDT), photosensitizers, and chemotherapeutics. Besides brief descriptions of chemotherapeutics’ classification and of PDT mechanisms and limitations, several examples of nanosystems endowed with different responsiveness (e.g., acidic pH and reactive oxygen species) and peculiarity (e.g., tumor oxygenation capacity, active tumor targeting, and biomimetic features) are described, and for each drug combination, in vitro and in vivo results on preclinical cancer models are reported.

**Abstract:**

The widespread diffusion of photodynamic therapy (PDT) as a clinical treatment for solid tumors is mainly limited by the patient’s adverse reaction (skin photosensivity), insufficient light penetration in deeply seated neoplastic lesions, unfavorable photosensitizers (PSs) biodistribution, and photokilling efficiency due to PS aggregation in biological environments. Despite this, recent preclinical studies reported on successful combinatorial regimes of PSs with chemotherapeutics obtained through the drugs encapsulation in multifunctional nanometric delivery systems. The aim of the present review deals with the punctual description of several nanosystems designed not only with the objective of co-transporting a PS and a chemodrug for combination therapy, but also with the goal of improving the therapeutic efficacy by facing the main critical issues of both therapies (side effects, scarce tumor oxygenation and light penetration, premature drug clearance, unspecific biodistribution, etc.). Therefore, particular attention is paid to the description of bio-responsive drugs and nanoparticles (NPs), targeted nanosystems, biomimetic approaches, and upconverting NPs, including analyzing the therapeutic efficacy of the proposed photo-chemotherapeutic regimens in in vitro and in vivo cancer models.

## 1. Introduction

Photodynamic therapy (PDT) is a therapeutic modality which uses visible light wavelengths, mainly in the red and near-infrared (NIR) regions, for the activation of photosensitizing molecules (PSs). In the presence of molecular oxygen, light energy absorption by PSs triggers a sequence of events, ultimately leading to the formation of singlet oxygen (^1^O_2_) and other cytotoxic reactive oxygen species (ROS) [1]. Thus, any kind of cells or tissues in which a PS accumulates and that has been exposed to appropriate light wavelengths can be killed by irreversible damages caused by oxidative stress. This phototherapeutic approach has been largely investigated and proposed for the treatment of several pathological conditions, even if PDT is mainly applied in the oncology field for the treatment of selected types of solid tumors [2]. The first clinical studies of PDT in humans date back to the 1970s, while regulatory approval of PDT for the treatment of certain types of cancer dates back to the 1990s [3,4,5]. Many clinical trials have been completed and others are still ongoing, but PDT remains an underused modality for treating solid tumors, compared to conventional radiotherapy, surgery, or chemotherapy [6,7]. However, PDT is largely used in dermatology for the removal of non-melanoma skin cancers and the treatment of precancerous conditions such as actinic keratosis, being minimally invasive and with therapeutic efficacy comparable to surgery [8,9].

Limited light penetration into deep-sited tumor masses and poor oxygen availability can impair the efficacy of PDT. Even under this circumstance, its selectivity and absence of important side effects, if compared to chemotherapy, continues to stimulate the research of innovative approaches to overcome limitations, while fully exploiting PDT advantages. Despite recent successes in the clinical application of PDT, chemotherapy continues to remain the therapy of choice for the treatment of most cancers due to its high potential to eradicate cancer cells. However, chemotherapy is a very aggressive treatment with severe side effects. For this reason, current research in the oncology field is focusing on developing combinations of PDT and chemotherapy, with the aim to achieve a synergistic effect between the two therapies. These multi-therapeutic strategies could provide a way to reduce the recommended doses of chemotherapeutics, while at the same time overcoming the limitations of PDT. The advantages derived from this combinatorial approach can be better exploited by adopting strategies for delivering combinations of drugs selectively into tumor, concomitantly minimizing undesired side effects in normal tissues. Recent studies are mainly directed toward the design and realization of smart nanocomplexes and nanoplatforms to deliver PSs in combination with additional anticancer agents to optimize the efficiency and selectivity of drugs uptake into malignancies and to potentiate the overall anticancer responses. 

The aim of this review is to briefly introduce the basic principles and mechanisms of action of PDT, as well as the most employed chemotherapeutic categories, before giving an overview of the recent literature on in vitro and in vivo preclinical studies of chemo-PDT nanosystems, highlighting the important role of PDT when combined with chemotherapeutic regimens. 

## 2. Basic Principles and Mechanisms of Photodynamic Therapy

As anticipated, PDT is a strategy for treating tumors based on three components (light, PS, and molecular oxygen), neither of which is per se harmful to cells, but whose interplay triggers the formation of ROS that, above a given threshold, causes severe/lethal cell damages. The PDT starts with a systemic administration of the PS (or topical in the case of skin cancers); after a selected time, which allows the PS accumulation in the neoplastic lesion, the tumor mass is irradiated with light wavelengths absorbed by the PS, leading to the production of ROS. Commonly used PSs produce mainly ^1^O_2_, whose lifetime in biological environments is ~4 µs, with a corresponding diffusion radius in the tissue of no more than ~220 nm [10,11]. This implies that the photooxidative stress induced by PDT remains confined to the cells and tissue in which the PS has been accumulated and activated, thus conferring intrinsic selectivity to the therapy. Most of the PSs are macrocyclic molecules such as porphyrins or their derivatives, such as chlorins, bacteriochlorins, and phthalocyanines. All these molecules absorb light in the red and far-red regions of the visible spectrum, the so-called “PDT therapeutic window” (660–800 nm). In this spectral range, tissues are more transparent due to the limited light absorption of endogenous chromophores, thus allowing deeper light penetration [1]. 

ROS produced during PDT treatment are harmful to cells and organisms, because they oxidize a variety of biological components, with proteins and membrane lipids being the major targets [12]. Because of the short diffusion capacity of ROS, the primary damaged cellular sites are determined by the intracellular localization of the PS during light irradiation, which in turn depends on the physico-chemical features of the PS. In general, hydrophobic PSs mainly localize in the intracellular membranes of endoplasmic reticulum (ER), Golgi apparatus and mitochondria, while the hydrophilic ones are mainly found in endosomes and lysosomes [13,14]. However, the intracellular localization of a PS is determined also by additional parameters, such as incubation time and cell type [15,16]. Depending on the PS localization and on the primary sites of photodamage, different cellular responses can be triggered by PDT. In particular, if the oxidative insult exceeds the cellular capacity to restore homeostasis, cell death can occur mainly via necrosis, apoptosis, or autophagy [17,18] or via less known PDT-induced cell death pathways, such as necroptosis [19,20], ferroptosis [21], and parthanatos [22,23]. It has been well characterized that high PSs and light doses cause heavy cellular damages and induce accidental necrosis, similar to PSs that are associated with the plasma membrane, which cause oxidative stress to proteins and phospholipids. This results in rapid loss of membrane integrity and subsequent cell lysis [15,24]. On the contrary, low PDT doses and PSs localized in mitochondria, ER, or lysosomes lead to apoptosis, which is induced by direct damages to the anti-apoptotic proteins Bcl2 and Bcl-xL, without affecting the pro-apoptotic protein Bax [25]. Damage-associated molecular patterns (DAMPs or alarmins) are released by or exposed on the surface of dying cancer cells. DAMPs are perceived as “alarm signals” and stimulate a specific anti-tumor response to the PDT-treated tumor as well as distal untreated metastasis [26,27]. Thus, PDT can inhibit tumor growth by the direct killing of cancer cells through one of the mechanisms outlined above and subsequent activation of innate and adaptative immunity [28,29,30]. 

PDT can also damage and affect the functionality of components of the tumor microenvironment (TME), which often plays a fundamental role in favoring cancer cell proliferation, tumor progression, and development of drug resistance [31]. As an example, disorganized and hyperpermeable tumor vasculature sustains rapid cancer cell proliferation with oxygen and nutrients supply. Tumor vessels are important targets of PDT, as they are easily affected by oxidative stress, thus resulting in vessel constriction, damages to endothelial cells, thrombi formation, and ultimately complete stasis of the blood flow, leading to severe hypoxia and tumor necrosis. In general, with PDT protocols based on short drug-light intervals, in which light is delivered to tumor when high PS levels are still circulating in the bloodstream, the vasculature represents the main target of the treatment, while cancer cells are killed indirectly through oxygen and nutrient deprivation [32].

## 3. Technological Approaches to Overcoming PDT Limitations

PDT is a minimally invasive and promising therapeutic modality for the treatment of localized and superficial tumors, notwithstanding several limitations, especially when considering deep-seated tumors, which might contribute to the scarce success of PDT in becoming a first-line treatment. It is often highlighted that PDT is selective since the oxidative stress induced by PS activation is confined to the irradiated area. Nevertheless, patients treated with PDT need to avoid unintentional exposure of skin and eyes to sunlight or artificial intense light. In fact, PSs accumulation in these organs is responsible for persistent photosensitivity [33,34]. This is one of the most serious PDT side effects and various strategies, exploiting passive and active mechanisms of targeting, which have been explored for increasing selective accumulation of the PSs in tumors. In this overview, the development of PS conjugates or PS-loaded delivery systems helps to maximize the anticancer effects, while decreasing unwanted photosensitivity [35]. Indeed, PSs have been conjugated to various types of carriers and/or targeting molecules, ranging from small ligands (e.g., folic acid and biotin), peptides, oligonucleotides, antibodies/antibody fragments, and hydrophilic polymers to modulate their physico-chemical properties. In addition to the above-mentioned strategies, nanotechnological approaches allow the physical entrapment or the covalent conjugation of PSs to nanosystems. Because of their size, nanosystems can extravasate from tumor blood vessels and accumulate preferentially into the TME, due to the enhanced permeability and retention effect (EPR). Considering their modularity in terms of chemical functionalization, nanosystems can be conjugated with ligands and, therefore, selectively internalized by cancer cells overexpressing specific receptors [36,37].

Moreover, PDT effectiveness highly depends on the homogeneous distribution of PSs in the tumor [38]. As for many anticancer drugs, the uptake of PSs in cancer cells decreases with the increasing distance from vasculature, thus increasing the survival chances after PDT treatment of cells located far from the blood vessels [39]. Approaches to increasing PSs penetration in the tumor site exploit the tumor-homing and tumor-penetrating properties of several peptides, generally indicated as “tumor penetrating peptides” [40]. For example, the most studied tumor penetrating peptides is iRGD, a 9-aminoacids cyclic peptide [41]. It has been shown that conjugation of iRGD to different PSs or PS-loaded nanocarriers increases the efficiency of uptake and penetration in in vitro 3D tumor models (multicellular tumor spheroids), as well as in in vivo human and murine tumor models [42,43,44].

The penetration of light into tissues can also be a limiting factor for the success of PDT treatments. Indeed, visible light wavelengths traditionally used in PDT achieve a maximum tissue penetration of ~12 mm. On this basis, the use of PDT to treat deep-seated tumors could result in insufficient or incomplete eradication of the mass [45]. Interstitial PDT, in which light is delivered intratumorally with optical fibers, represents the first and most obvious approach to overcoming this limitation [46,47]. Another workaround addressing this limitation is the development of PSs with absorption ranges shifted toward NIR wavelengths. Among the PSs approved or under evaluation for clinical use, TOOKAD exhibits efficient absorption at ~760 nm, resulting in enhanced PS activation in deeper tissue. Preclinical studies on human prostatic small cell carcinoma xenografted in mice indicated that TOOKAD-PDT induced necrosis to a depth of ~1.3 cm [48].

Tissues have a maximum transparency at light wavelengths falling in the window between 780 and 950 nm. However, PSs are unable to produce significant amounts of ^1^O_2_, as the energy content carried by these wavelengths is exceedingly low [49]. Thus, two-photon excitation has been proposed for PS activation using low-energy wavelengths [50,51]. Considering that most available PSs are not suitable for two-photon excitation, new PSs have been designed for their activation with a femtosecond pulsed laser at 800 nm. It has been demonstrated that they could induce tumor regression to a depth of ~2 cm [52,53]. Even in the overview of exploiting low-energy wavelengths, several nanotechnology-based approaches have been recently proposed. As an example, gold nanoclusters coated with dihydrolipoic acid were efficiently activated by two-photons absorption with an 800 nm femtosecond laser. This resulted in an efficient growth inhibition of hepatocellular carcinoma-derived xenografts, thanks to the formation of O_2_^−^ instead of ^1^O_2_. It was suggested that these nanoclusters can also induce cytotoxic effects in the hypoxic regions of the tumors [54,55]. Similarly, upconverting nanoparticles (UCNPs) offer another possibility to activate PSs using low-energy wavelengths. UCNPs doped with rare-earth ions possess unique properties to absorb NIR wavelengths and emit fluorescence in the visible region [56,57,58]. PSs can be directly conjugated to the surface of the UCNP or partitioned into a coating layer [59]. As an example, it has been reported that visible light-absorbing PSs can be entrapped into a lipid bilayer coating the UCNPs (NaYF4:20%Yb3^+^/2%Er3^+^). Irradiation with a 980 nm laser induced cancer cells killing both in vitro and in intratumorally injected mice [60]. Another work reported that Janus nanocomposites, formed by an upconverting nanocrystal head and a mesoporous silica body loaded with chlorin e6 (Ce6), induced efficient antitumoral effects in vitro and in vivo under a 980 nm irradiation. Alternatively, persistent luminescent NPs have been proposed as another possible solution for delivering light to the tumor site, allowing PS activation without external light sources [61]. Persistent luminescence can be triggered by NP activation with UV and 808 nm light or by chemiluminescent reactions occurring in the TME [62,63]. Thus, two-photon excitation, UCNPs and persistent luminescent NPs appear promising for addressing the problem of light penetration. However, these approaches still need to solve problems related to the small volume of irradiated tumor and overheating causing normal tissue damage [64]. 

Tumor oxygenation is another key point for PDT outcomes, and it depends on the degree of vascularization. Blood vessels in solid cancers are highly disorganized compared to normal tissues, and they do not provide sufficient blood and oxygen supply, leading to the formation of hypoxic regions in larger neoplastic masses [65]. Being strongly dependent on O_2_ concentration, PDT treatment is strongly attenuated in hypoxic regions of tumors. Indeed, a partial pressure of O_2_ in certain tumor regions falls under 5 mmHg (7 µM), while the efficiency of PDT begins to decline when the O_2_ pressure drops below 15–35 mmHg [66,67]. Cancer cells located in these hypoxic cores are hardly affected by PDT [68]. Based on these considerations, various strategies have been proposed for increasing O_2_ concentration in tumors [69]. Early preclinical and clinical studies showed higher efficacy of PDT delivered under hyperoxygenation conditions with respect to normoxia [70,71]. However, the intrinsic side effects of hyperbaric oxygen therapy, as hyperoxic seizures and barotraumas, stimulated the development of different strategies to alleviate tumor hypoxia. Recent studies have proposed different nanoassemblies or artificial red blood cells containing natural or artificial O_2_ carriers, together with PSs, for a more tailored delivery of the two agents to the tumor. Hemoglobin was loaded into liposomal vesicles or embedded into red blood cell membranes for delivering O_2_ in the TME [72,73,74]. As an alternative, perfluorocarbons (PFC) and O_2_ have been co-loaded with PSs in liposomes or other nanocarriers with the aim of improving tumor oxygenation [75,76,77,78]. Other approaches exploit the unique features of the TME, such as high H_2_O_2_ concentration. For example, catalase can be exploited to catalyze H_2_O_2_ decomposition into O_2_. The enzyme has been physically or covalently co-loaded with PSs in polymeric, organic, or inorganic NPs functionalized with targeting elements. In vivo studies showed that its delivery in tumors alleviated hypoxia, decreased the expression of hypoxia-inducible factor 1-α (HIF-α) and increased PDT efficacy [79,80,81]. Considering instability and proteases degradation issues of catalase, other authors have proposed nanomaterials with catalase-like characteristics (nanozymes) such as platinum, manganese dioxide, or Prussian blue NPs. Once assembled, PS-containing nanocomposites improved tumor oxygenation and PDT efficacy [82,83,84,85]. Other approaches alleviate hypoxia by decreasing oxygen consumption by tumor cells. In fact, inhibitors of the mitochondrial respiratory chain as metformin (Met), currently used for treating type II diabetes, have been proposed in combination with various PSs, showing that the proposed combination reduced hypoxia and improved PDT performance [86]. 

In any case, while several different and fruitful strategies have been investigated as the possibilities to overcome the inherent drawbacks of PDT, it appears clear that the beneficial PDT therapeutic effects can be better exploited in combination with some conventional chemotherapeutics, as will be described in detail in the following paragraphs.

## 4. Overview of Main Clinically Approved Chemotherapeutics

In chemotherapy, potent cytotoxic drugs are used to disrupt the way cancer cells proliferate, grow and divide. Therefore, chemotherapy is most effective in killing cancer cells as they replicate faster than healthy cells, and it is precisely during replication that most cytotoxic drugs act. In this paragraph, the main categories of clinically approved chemotherapeutics and their toxicity mechanisms will be briefly described to contextualize the combination regimes proposed in association with PDT and NP-based drug delivery systems.

### 4.1. Antibiotics

This class of anticancer drugs is derived from natural anthracycline compounds produced by microorganisms of the species *Streptomyces*. It is mainly represented by doxorubicin (DOX) and daunorubicin (Dau). The primary mechanisms of action of these cytotoxic agents are as follows:

- DNA intercalation and production of single- and double-strand breaks;
- Oxidative stress caused by the generation of important concentrations of free radicals;
- Inhibition of topoisomerase II and disruption of enzyme-mediated DNA repair mechanisms.

Although being widely used for the treatment of several types of tumors, such as ovarian, breast, lung, and lymphomas, anthracycline antibiotics suffer from serious heart-related side effects [87].

### 4.2. Antimitotic Agents

Antimitotic agents can be divided in two main groups:

- Taxanes are a class of drugs derived from diterpenes produced by plants of the genus *Taxus*. They stabilize microtubules polymerization by binding to a specific domain found in β-tubulin. Clinically approved taxane agents are paclitaxel (PTX), docetaxel (DTX), and cabazitaxel (CBZ).
- Vinca alkaloids are derived from *Catharanthus roseus*. They induce the destabilization of the polymerization process of microtubules by binding to the interface between α- and β-tubulin.

Antimitotic agents induce cell cycle arrest by mitotic spindle interference caused by the disruption of microtubules polymerization dynamics. Subsequently, cells undergo mitotic arrest and cell death through mitotic catastrophe. Both taxanes and Vinca alkaloids suffer from solubility issues and produce severe side effects on the immune system (i.e., neutropenia) [88].

### 4.3. Platinum-Based Chemotherapeutics

Platinum-based drugs belong to the class of alkylating agents, being highly reactive molecules that react with the most of the intracellular biocomponents. For these reasons, the mechanism of action of these drugs is due to a complex sequence of events, even if the main mechanism determining mortality of cancer cells is the alkylation of nucleic acid at the guanine residues. DNA alkylation produce single- and double-strand breaks, which ultimately lead to interstrand and intrastrand DNA crosslinking, resulting in replication and transcription arrest. Several drugs belong to the class of alkylating agents, but the most used in therapy are platinum complexes, such as cisplatin, carboplatin, and oxaliplatin. 

The major limitation of these drugs is the suppression of the bone marrow functionality, causing anemia, leukopenia, and thrombocytopenia. Another important disadvantage is their low therapeutic indices [89].

### 4.4. Topoisomerases Inhibitors (TIs)

Basically, topoisomerases inhibitors (TIs) are a class of anticancer compounds able to kill neoplastic cells by interrupting DNA replication by interfering with topoisomerase I (TOPI) or topoisomerase II (TOPII) enzymes, which role deals with the creation of single- or double-strand DNA breaks, respectively. 

Since TOPI works by forming a transient complex with DNA intermediates (e.g., the cleavage complex) to create the break and then repromote DNA ligation after replication, TOPI inhibitors stabilize the cleavage complex and maintain the strand breaks [90]. Among clinically approved TOPI inhibitors, the natural compound camptothecin (CPT) and its synthetic derivatives such as topotecan (TPC), irinotecan (IRI), and belotecan (BLT) are widely employed for treating various cancer types, even if they suffer from scarce water solubility, low biocompatibility, and toxic side effects and induce resistance [91]. Of note, non-CPT compounds (e.g., indenoisoquinoline, phenanthridines, and indolocarbazoles) are investigated, since they display improved chemical stability, reduced reversibility, and shortened infusion times with respect to CPT derivatives [90,92].

A huge list of compounds belongs to the TOPII inhibitors [93], but the main classes comprise catalytic inhibitors (antibiotics belonging to the groups of coumarins and quinolones) and intercalating (e.g., DOX and derivatives) or non-intercalating (e.g., etoposide and teniposide) drugs. 

## 5. Drug Delivery Systems for PSs and Chemotherapeutics: An Overview of Recent Advances

As anticipated in Section 4, nanotechnology-driven approaches impose themselves even in the context of pharmacy and medicine, positively affecting anticancer drug delivery research. Indeed, the encapsulation of drugs/drug combinations in nanometric drug delivery systems (DDSs), made of several inorganic or organic materials [94], significantly ameliorates therapeutic indices due to increased drugs availability, improved pharmacokinetics, and tumor targeting capacity. Plenty of examples of DDSs for PSs and chemotherapeutics have been published in the literature in the last two decades; most papers included efficacy investigations at the preclinical level, while clinical studies on humans are very restricted. Nevertheless, in this section, we described several examples of DDSs (summarized in Table 1) developed for the simultaneous delivery of PSs and chemodrugs, to highlight how the presence of the nanosystem and the synergistic interaction between the drugs/therapies significantly improve the overall anticancer effect.

### 5.1. Combination of PSs with Antibiotics

Among antibiotics, DOX, which belongs to the class of anthracyclines, is one of the most used chemotherapeutics for both hematopoietic and solid tumors [114]. Accordingly, our literature survey on NP-mediated delivery of antibiotics with PSs revealed the predominant use of DOX among other antibiotic drugs; consequently, in the present subsection, all described nanoformulation examples are restricted to DOX. 

Notwithstanding the high anticancer potential, clinical use of DOX is associated with several adverse effects such as severe cardiotoxicity, which depends on the administered dose and often imposes the modulation of the therapy [115]. Therefore, the combination of DOX-based chemotherapy with other anticancer modalities may relieve collateral effects by lowering doses, while preserving treatment efficacy. Indeed, the combination of DOX with PDT has been widely investigated, using various types of nanosized carriers, designed to fully exploit the potential additive/synergistic effects of this combined regimen [116,117,118], offering a way to address the limitations described above.

**Scheme 1:**
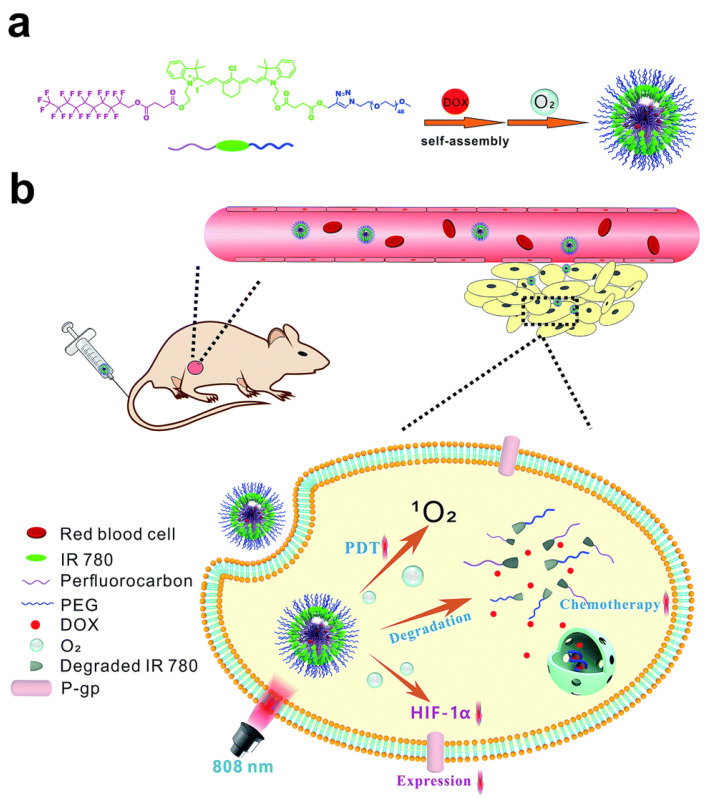
Schematic representation of the rationale and of the features of F/DOX NPs developed by Yang and co-workers. The figure is reprinted from [95]. In (**a**) are pictured the steps of F/DOX NPs synthesis, while in (**b**) the authors schematize how their drug delivery system could enhance tumor therapy outcome by combing the photodynamic ef-fect and DOX chemotherapy.

For example, Yang et al. [95] developed a nanocarrier for chemo-PDT with tumor hypoxia-relieving features using a conjugate formed by a hydrophobic perfluorocarbon as an oxygen carrier (F), an NIR-absorbing PS (IR780), and a hydrophilic PEG chain. The so-called F-IR780-PEG conjugate self-assembled into F-micelles and encapsulated DOX and O_2_ in the hydrophobic core, thus forming the F/DOX NPs. It was observed that under an 808 nm laser irradiation, IR780 caused cytotoxic effects, and the generation of ROS promoted its photobleaching, which ultimately triggered NPs disassembly and controlled DOX release (see Scheme 1).

Following laser irradiation in vitro, MCF7 breast cancer cells treated with F/DOX NPs exhibited remarkable synergistic cytotoxicity compared to free DOX or F micelles (PDT only), under both normoxia and hypoxia. Moreover, F/DOX NPs uptake in vivo was confined within the tumor mass, very likely due to the EPR effect. Growth of irradiated tumors of mice injected with F micelles was strongly affected compared to that of mice treated with micelles not containing perfluorocarbons, indicating the fundamental role of F-micelles in carrying oxygen. Furthermore, F/DOX NPs treatment resulted in a consistent downregulation of HIF-1α protein and P-glycoprotein, with consequent alleviation of hypoxia and DOX resistance.

As mentioned above, another major problem related to PDT is persistent photosensitivity after treatment. The unique features of the TME (e.g., low pH and reduced environment) can be exploited to achieve a spatio-temporal controlled release of PSs and chemotherapeutics. The wide selection of biomaterials provides resources to develop NPs bioresponsive to the TME [119,120]. The mild acidic conditions of the extracellular TME (pH 6–7), as well as the intracellular acidic pH of endosomes and lysosomes (pH 5–6), are largely exploited to trigger drug release, thanks to the use of ionizable chemical groups, acid-labile chemical bonds, pH-sensitive peptides, or pH-responsive polymers for NP synthesis.

A smart nanocarrier was proposed by Tian et al. [96], by the use of a pH-sensitive polymer poly(2-(diisopropylamino)ethyl methacrylate) (PDPA) for the fabrication of self-assembled tetraphenyl porphyrin (TPP)- and DOX-containing Janus macromolecular brushes (denoted by the authors as Brush 1 NPs). Once in the TME or in cancer cells, the acidic pH (<6.3) induced the polymer switched from hydrophobic to hydrophilic, thus permitting its exploitation in controlled drug release [121]. The presence of TPP covalently bound to the polymer improved the loading of DOX by π−π interactions and showed high drug loading and stability in PBS (pH 7.4), where only 13% of DOX was released in 20 h. On the contrary, under acidic pH (6.5 and 5.5), DOX release was enhanced to 40% and 69%, facilitated by PDPA chains protonation and NP disassembly, respectively. Importantly, the collapsed PDPA chains increased the distance between TPP molecules, suppressing the aggregation-caused quenching effect and enhancing singlet oxygen production [122]. The intracellular uptake and distribution of TPP and DOX loaded in the NP formulation were studied in vitro in 4T1 mouse triple-negative breast carcinoma cells, where the intensity of the intracellular signal of TPP delivered by Janus NPs was stronger than that of free TPP. Accordingly, Brush 1 NPs treatment following irradiation resulted in the most significant reduction in cell viability, compared to control NPs not endowed with TME responsiveness or containing drugs singularly. NIR fluorescence imaging in 4T1 tumor-bearing mice indicated that Brush 1 NPs reached a maximum accumulation after 24 from the i.v. injection. Moreover, the fluorescence signal persisted at least up to 48 h post-injection, indicating effective intertumoral retention due to the EPR effect. As expected, based on the biodistribution studies, tumor irradiation with a 655 nm laser light resulted in an improved antitumor effect in Brush 1 NPs-treated mice. 

Several studies have been focused on the development of ROS-responsive NPs able to disassemble and release the carried drugs exclusively in the presence of elevated ROS levels, like those found in the TME. Among ROS-responsive linkers, the thioketal (TK) appears to be ideal for conjugating drugs in NPs thanks to its stability in normal biological conditions, while undergoing rapid degradation in the presence of ROS [123]. 

**Scheme 2:**
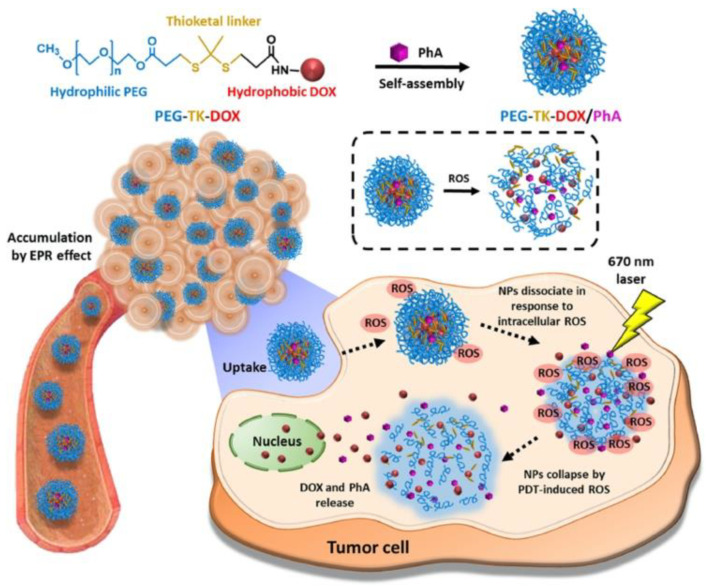
Schematic representation of the mechanisms of action of PEG-TK-DOX/PhA NPs. The figure is reprinted from Kim et al. [97].

Kim et al. [97] reported the conjugation of DOX to PEG using a TK linker (PEG-TK-DOX conjugate). When in an aqueous medium, the conjugate self-assembled entrapping the PS pheophorbide A (PhA) in PEG-TK-DOX/PhA NPs. A spatiotemporally controlled release of DOX and PhA was realized in a “cascade-like manner”, triggered firstly by endogenous ROS and secondly by PDT-produced ROS, leading to the complete release of both drugs as illustrated in Scheme 2. CT26 mouse colon cancer cells were treated with the free drugs, their combination, and PEG-TK-DOX/PhA NPs, and the drug combination led to a synergistic cytotoxic effect, according to the combination index (CI) analysis [124]. This data suggested that the improved NPs cellular toxicity is due to the combined effect of the ROS produced during PhA irradiation, which enhanced DOX release after the dissociation of the TK linker. The in vivo accumulation of PEG-TK-DOX/PhA NPs after i.v. injection in tumor-bearing mice was higher in the irradiated group, thereby confirming the important role of ROS in nanoparticle disassembly. In addition, the histological analysis showed myocardial damage in mice treated with free DOX, while no damage was evident in mice treated with PEG-TK-DOX/PhA NPs. Importantly, these observations highlighted that these self-assembled and bioresponsive NPs may represent a valid drug formulation to reduce the cardiotoxic effects of DOX. 

In addition to NPs exploiting passive targeting, several authors have proposed drug delivery systems endowed with active targeting properties for improving the selectivity of the chemo-PDT regimen. 

Zhang and collaborators [98] developed a pH-responsive nanoplatform (RGD-NPs/Ce6) carrying an RGD-targeting element and loaded with Ce6 and DOX prodrug (CRGDK-PEG-DOX). pH sensitivity is due to the presence of a Schiff base in the prodrug conjugate. It was reported that less than 10% of the drugs were released from RGD-NPs/Ce6 at pH 7.4, about 30% of the drugs were released from RGD-NPs/Ce6 at pH 6.5, and almost 70–80% of the drugs were released from RGD-NPs/Ce6 at pH 5.0 within 24 h. The specific and receptor-mediated internalization of NPs was demonstrated in breast cancer cells MDA-MB-231 and MCF-7 with high and low expression of Neuropilin-1 receptor (NRP-1), respectively. RGD-NPs/Ce6 accumulated more efficiently in MDA-MB-231 cells compared to NPs/Ce6, while significant differences were not found in MCF-7 cells, confirming the specific recognition of the RGD peptide by the cells. As expected, higher ROS levels were generated in MDA-MB-231 cells, and RGD-NPs/Ce6 showed the strongest antitumor efficacy. The in vivo biodistribution showed a longer blood circulation and a higher intratumoral accumulation of NPs, where RGD-NPs/Ce6 showed a stronger fluorescence intensity thanks to the NRP-1 receptor-mediated endocytosis. A similar strategy was reported by He et al. [125], in which Ce6/Dox@NPs-cRGD NPs were conjugated to the cyclo(Arg-Gly-Asp-d-Phe-Cys) peptide, and it was shown that this cyclic peptide exhibited a selective targeting capability for the α_ν_β_3_ receptor [126]. 

Another possible strategy to achieve active targeting is represented by hyaluronic acid-conjugated (HA) nanoplatforms, which can be directed towards CD44^+^ cells. He et al. [99] realized a targeted nanovehicle able to release drugs selectively to cancer cells in response to hypoxia. These authors prepared DOX-loaded NPs (HC/PN/DOX) by self-assembling of polyethylenimine-nitroimidazole (PEI-NI) NPs with Ce6-HA (HC). The hydrophobic NI group could be converted into the hydrophilic 2-aminoimidazole (AI) group under the hypoxic conditions, triggering NPs dissociation and DOX release. The in vitro studies on NPs uptake in CD44^+^ mouse Lewis lung carcinoma (LLC) cells confirmed NP targeting capability through the recognition of HA and consequent CD44-mediated endocytosis. HC/PN/DOX NPs displayed strong anticancer effects, which was substantially diminished upon incubation with vitamin C, a strong ROS scavenger, supporting the evidence that light-triggered hypoxia in cancer cells could promote DOX release. The in vivo biodistribution of NPs was determined using 1,1-Dioctadecyl-3,3,3,3-tetramethylindotricabocyanine iodide (DiR)-loaded NPs: the accumulation of HC/PN/DiR NPs was 2.9-fold higher than free DiR, suggesting that CD44-targeting facilitated tumor accumulation. Given the crucial role of hypoxia in NP disassembly, tumor oxygen levels were assessed by in vivo photoacoustic imaging before and after PDT. The blood oxygenation status (sO_2_) dramatically decreased from 15.5% (before treatment) to 2.1% (4 h after irradiation), demonstrating the occurrence of hypoxia in PDT-treated tumors. As expected, the tumor growth was remarkably inhibited in mice treated with HC/PN/DOX NPs, with a consequent prolonged survival of the animals.

In recent years, biomimetic NPs have been proposed as carriers for increasing bioavailability and selective delivery, accumulation, and penetration of anticancer drugs in solid tumors [127,128]. Biomimetic NPs are composed of a synthetic core made of inorganic/organic materials with multiple functionalities and coated with a layer of cell membranes mimicking the properties and functions of the source cells, such as immune escape, prolonged circulation in the bloodstream, and tumor-targeting capability. 

Biomimetic NPs were used by Jin et al. [100] against metastatic triple-negative breast cancer (TNBC). NPs core was formed by upconverting NPs co-loaded with Rose Bengal (RB) and the ROS-sensitive polymer polyethylene glycol−thioketal−doxorubicin (PEG-TK-DOX, named PTD) and coated with cancer cell membranes (CMs). The membrane camouflaged CM@UCNP-RB/PTD NPs were activated using a 980 nm NIR light and resulted in visible luminescence emissions, thus triggering production of ROS by RB and, consequently, DOX release. The CM coating possessed an epithelial cell adhesion molecule (EpCAM), N-cadherin and CD47, which are crucial for homologous adhesion to cancer cells and immune escape [129]. The in vitro uptake of CM@UCNP-RB/PTD in homologous 4T1 cancer cells demonstrated the efficacy of this targeting strategy, while lower accumulation was detected in normal cells and non-homologous cancer cells. Following irradiation, DAMPs, calreticulin expression, and ATP release were detected, indicating the occurrence of immunogenic cell death (ICD), which stimulated the in vitro maturation of dendritic cells, suggesting the possibility to elicit an antitumor immune response. In vivo tumor growth was strongly inhibited (80.5%) following CM@UCNP-RB/PTD treatment, validating the efficacy of the CM coating.

### 5.2. Combination of PSs with Antimitotic Agents

As mentioned in Section 4, PTX and DTX are clinically approved anticancer agents that belong to the taxane family. They are commonly used as single drugs or in combination with other chemotherapeutics, for the treatment of various types of cancers including breast, ovarian, lung, prostate, and AIDS-related sarcoma. By interfering with microtubules dynamic and arresting the cell cycle in the G2/M phase, PTX and DTX exhibit potent anticancer activity, even if their efficacy is associated with several important adverse side effects and the development of resistance. Various strategies are explored in order to preserve their high antitumoral efficiency while reducing side effects. Even in this context, the development of delivery systems and the combination with other treatment modalities, including PDT, has received particular attention [130]. 

PTX and DTX are hydrophobic drugs, and the development of NP-based formulations is crucial not only to overcome solubility issues but also to circumvent drug resistance and modulate their co-delivery with additional anticancer agents. Indeed, it has been shown that taxanes are able to induce aggregation of several proteins, including human serum albumin (HSA), promoting the formation of protein-based NPs which serve as natural carriers for taxanes and additional therapeutics/diagnostic agents. 

**Scheme 3:**
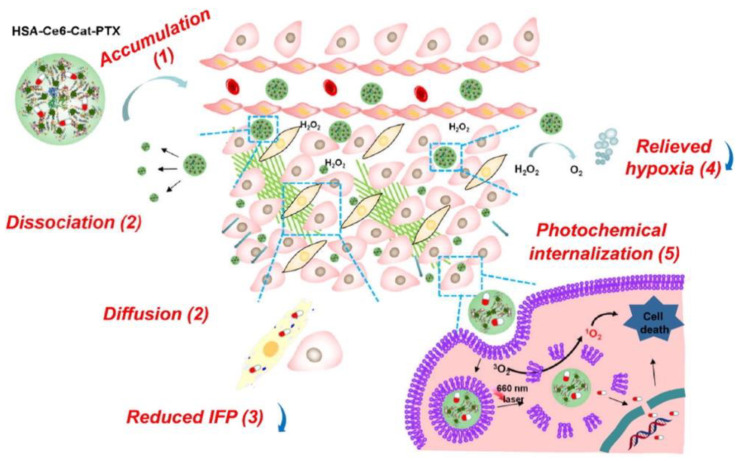
Schematic representation of the mechanisms of action of HSA-Ce6-Cat–PTX NPs. The figure is reprinted from Chen et al. [101].

As an example of protein-based delivery systems for chemotherapy and PDT, Chen et al. [101] developed multifunctional HSA NPs loaded with the enzyme catalase (Cat) and co-delivering PTX and the PS Ce6. The size of NPs obtained by PTX-induced co-assembly of HSA and Cat (HSA-Ce6-Cat–PTX) was in the range 50–100 nm and was preserved during the circulation in the blood stream, guaranteeing effective tumor accumulation via the EPR effect. It was also observed that because of dilution effects these NPs dissociated into particles smaller than 20 nm within the tumor, allowing deep penetration into the malignancy. Interestingly, when Cat was co-loaded in NPs, it was not susceptible to decomposition by proteases during circulation in the blood stream and preserved its catalytic activity, thus decomposing H_2_O_2_ to produce O_2_ and decreasing hypoxia for optimizing the PDT antitumor activity. In vitro uptake studies on 4T1 cells showed a greatly improved accumulation of Ce6 after being incorporated into protein-based NPs with respect to free Ce6 and consequently enhanced PDT-induced toxicity. Compared to chemotherapy alone with HSA-Ce6-Cat–PTX (cells not irradiated) or PDT alone with HSA-Ce6-Cat NPs (cells irradiated with a 660 nm light), the combined treatment with HSA-Ce6-Cat–PTX NPs and light showed a clear synergistic effect. Ex vivo imaging of tumors and major organs collected from mice i.v. injected with the various formulations confirmed a higher tumor uptake of HSA-Ce6-Cat–PTX due to prolonged blood circulation. Importantly, the fluorescence signals of Ce6 loaded in HSA-Ce6-Cat–PTX NPs in the analyzed tumors were also located far from blood vessels, indicating diffusion and penetration favored by dissociation into small protein complexes. By contrast, Ce6 fluorescence signals of cross-linked HSA-Ce6-Cat NPs were mostly co-localized with tumor blood vessels, demonstrating that large particles were unable to diffuse through the tumor extracellular matrix. Photoacoustic (PA) imaging of 4T1 tumors before and 24 h post-injection of HSA-Ce6-Cat–PTX NPs showed an increase in the oxyhemoglobin level in the tumors, where nearly no hypoxia markers (anti-pimonidazole and HIF-1α) were found, confirming the oxygenation favored by Cat activity. Thus, as shown in Scheme 3, the authors have adopted a very simple preparation method based on drug-induced proteins assembly to obtain NPs with multiple functions that could be utilized for effective in vivo combination therapy. 

In the context of nanomedicine and especially using taxanes, a quite different approach to the development of smart drug formulations has been gaining increasing attention: it is based on the synthesis of prodrug-based nanocarriers avoiding the use of template materials for NPs production [131,132,133].

Thus, even in the overview of simplifying NP architecture and synthesis procedures, very recently, Moret et al. [102] reported a modified nanoprecipitation method which exploits PTX in the form of a tumor bioresponsive prodrug (e.g., PTX_2_S, formed by two molecules of drug linked by a thioether linker responsive to high concentrations of GSH and/or ROS) to form self-assembled micelles incorporating the PS pheophorbide A (PheoA). The reported one-pot synthesis procedure allowed the preparation of PheoA≅PTX_2_S NPs with impressive capabilities of 100% and 40% loadings of PTX and PheoA, respectively, without the use of additional exogenous materials to propose an ideal nanoplatform for combined chemo- and phototherapy. Indeed, the authors reported that, exclusively in a simulated TME (e.g., 10 mM GSH and 500 μM H_2_O_2_), NPs efficiently disassembled the drugs, while they remained stable in physiological conditions and preserved the PheoA-mediated capacity to efficiently produce ^1^O_2_ upon light irradiation. The bioresponsiveness of the proposed PTX-prodrug delivery system was verified also in vitro in normal (CCD-34Lu human lung fibroblasts) versus cancer cells (breast-derived MDA-MB-231 and ovarian-derived SKOV-3), finding that only micellar PTX_2_S scarcely affects the viability of normal cells, while significantly influencing those of both tumor cell lines, accordingly to an improved PTX release in the case of the reductive TME. In vitro combination therapy was assessed in cancer MDA-MB-231 and SKOV-3 cells by applying Chou and Talalay’s method and indicated that, even if the synergism between therapy was limited in both cell lines, a 30-fold PTX dose reduction was allowed in the ovarian cell line. 

The same group also reported on a substantially different approach to increase the targeted delivery of the same drug combination (PTX and PheoA) by exploiting endogenous HSA binding, thus promoting NPs/drugs uptake via the EPR effect plus HSA-mediated uptake [103]. Indeed, they proposed a micellar nanoplatform made by the spontaneous self-assembly in water of the PTX homodimer (PTX_2_S), PheoA, and a novel PTX bio-responsive PEGylated conjugate armed with an HSA-binding moiety (e.g., maleimide, MAL) (PTX-SS-PEG-MAL). The authors reported the formation of highly reproducible and monodispersed micelles with a size of 80 nm, with a MAL-PTX conjugate/PTX_2_S/PheoA ratio of 56%:32%:12% (*w*/*w*/*w*) for a final PTX/PheoA ratio of 2.5:1 (*w*/*w*), compatible with biological assays. Importantly, using a LC-MS-based assay, the capability of the MAL-conjugated prodrugs to effectively bind HSA in vitro was demonstrated, forming a stable adduct with a 94% affinity with respect to 4.6% of a BOC conjugate (e.g., a non-HSA-binding prodrug). In vitro investigations comparing the therapeutic efficacy of PTX prodrugs and PheoA loaded in MAL-decorated vs. BOC-decorated loaded micelles (and PTX_2_S micelles and free PheoA as controls) were performed in three different breast cancer cell lines (human MCF-7 and MDA-MB-231 and mouse 4T1) cultured. Monolayers or as three-dimensional spheroids showed an improved killing efficiency of MAL-micelles with respect to BOC micelles in the human cell lines, while it was comparable in the mouse-derived cells, notwithstanding a significantly higher intracellular uptake of the HSA-binding micelles in all the three cell lines. Indeed, 4T1 cells demonstrated to be extremely resistant to PTX treatment both in vitro and in vivo when implanted in BALB/c mice, in which a very poor tumor mass reduction was observed following MAL or BOC micelles treatment with respect to PTX or PheoA administered as a single treatment, notwithstanding the significant capacity of the MAL-decorated nanoformulation of reducing lung metastasis. 

ROS-responsive dimeric prodrugs were used also by Pei et al. [104] to realize a red blood cell (RBC) membrane-camouflaged NP. This NP was formed by an inner core containing a ROS-responsive PTX dimer with a thioketal linker (PTX2-TK) and the PS 5,10,15,20-tetraphenylchlorin (TPC) strongly associated to PXT prodrug by π−π stacking, finally coated with an RBC membrane. The “self-recognized” or “do not eat me” proteins of the RBC membrane conferred to the camouflaged NP’s capacity to escape from the RES capture. In fact, it was shown that the RBC membrane coating strongly inhibited the capture of RBC(M(TPC-PTX)) NPs by mouse RAW264.7 macrophages in comparison to uncoated M(TPC-PTX) NPs. Upon light irradiation, the integrity of the coating membrane was lost, and the NP content was released as documented by the re-localization of TPC fluorescence from endosomes/lysosomes of human cervical carcinoma HeLa cells to a more diffused distribution in the cell cytoplasm. Moreover, the authors observed that, at the TPC doses used for cytotoxicity tests in HeLa cells, PDT alone reduced the viability by less than 50%, while in cells incubated with PTX/TPC-containing NPs and irradiated, lethality was higher, indicating synergistic effects of combination therapy. The benefits brought by NP camouflaging with RBC membranes were evident in the in vivo pharmacokinetics and biodistribution studies which showed longer blood circulation of RBC(M(TPC-PTX) NPs than naked M(TPC-PTX) NPs as well as reduced uptake in the liver. Following i.v. injection, the concentrations of PTX2-TK in the tumor of mice that received RBC(M(TPC-PTX)) NPs were higher than in those treated with M(TPC-PTX) NPs over time and were 4.6-fold higher at 23 h post-injection, further demonstrating the importance of the membrane coating. In addition, immunofluorescence staining of the tumor vasculature highlighted that irradiation induced extravasation and deep tumor penetration of RBC(M(TPC-PTX)) NPs. The in vivo antitumor efficacy studies performed in HeLa tumor-bearing mice showed that chemotherapy and PDT alone could not eliminate tumors. On the contrary, remarkable reduction of the tumor volume was obtained with naked M(TPC-PTX) NPs plus a 638 nm laser irradiation, but the strongest antitumor effect was achieved with RBC(M(TPC-PTX)) NPs plus irradiation, due to their long blood circulation and preferential tumor accumulation. The histologic images of H&E-stained sections of the major organs showed no distinct cellular damages, indicating that RBC(M(TPC-PTX)) NPs exhibited excellent therapeutic effects and showed nonsignificant systemic toxicity. 

As already outlined in the previous sections, many investigations were focused on the realization of multifunctional delivery systems which are TME responsive and/or exploit active mechanisms of targeting. Considering the highly reductive characteristics of the TME and of tumor cells, Wang et al. [105] proposed redox-responsive hyaluronic acid (HA)-based NPs for targeted photodynamic therapy/chemotherapy co-delivering DTX and PS Ce6, taking a cue from previously developed NPs not endowed with bio-responsiveness [134,135]. These NPs are biocompatible, biodegradable, and non-immunogenic, because they are formed by a natural hydrophilic polymer that can be chemically modified to fabricate NPs with well-defined properties. In this work, HA molecules were modified with hydrophobic docosahexaenoic acid (DHA) and Ce6 via cysteamine (cys) as a connecting arm to obtain an amphiphilic HA derivative that could self-assemble in water into NPs with a core–shell structure and encapsulating DTX in the core. In the low reductive environment, the drug release from NPs was very slow, while the release rate increased significantly in higher reductive conditions, indicating responsiveness caused by rupture of the disulfide bond in the linking arms. The cellular uptake studies in MCF-7 cells indicated a higher uptake of Ce6 delivered with NPs with respect to free Ce6 as well as CD44 receptor-mediated endocytosis of NPs, considering that MCF-7 cells express CD44 that specifically bind HA. The cell cytotoxicity induced with PDT and DTX as single treatments and in combination were stronger with NPs than with free drugs and correlated with the intracellular uptake. DTX treatment blocked the cell cycle in the G2/M phase with all the formulations, but the largest fractions (97.33%) of cells in G2/M phase was found with the combination of PDT and DTX using NPs for their delivery. In vivo and ex-vivo fluorescence imaging studies with BALB/c mice bearing 4T1cells indicated that free Ce6 was quickly cleared from the body, while that encapsulated in NPs persisted especially in tumor and liver since a strong fluorescence was still detected at 24 h post-injection. The in vivo antitumor efficacy studies showed that, compared with chemotherapy or PDT alone, the combined treatment based on the co-delivery of DTX and Ce6 by NPs plus NIR light showed the best antitumor effect. Of note, the authors stated that the drawback of these HA-based NPs was the high accumulation in the liver, caused by elevated phagocytosis rate by Kupffer cells, based on which they also concluded that the same NPs could be considered for applications in liver targeting [136]. 

Nanoemulsions are versatile delivery systems characterized by a high solubilization capacity and stability allowing the administration of drugs by varying routes, including intravenous ones [137]. For the combination of PDT and chemotherapy, Chang et al. [106] realized a nanoemulsion constituted by a glyceryl trioctanoate oil core solubilizing PTX and porphyri-lipid forming a shell to stabilize the water/oil interface. The PLNE-PTX nanoemulsion was further stabilized with the incorporation of 5% mol DSPE-PEG2000. It was shown that PEGylation of PLNE-PTX nanoemulsion prolonged plasma circulation and enhanced accumulation in tumor. PLNE-PTX was rapidly internalized in KB cells in vitro: after 24 h of cell incubation, nanoemulsions were disrupted, and the monomeric porphyrin-lipid allowed fluorescence imaging and PDT efficacy. Laser irradiation with a 671 nm light following cell incubation with PLNE-PTX caused a decrease of cell viability that was enhanced by 7.5-fold and 3.9-fold compared to chemotherapy alone and PDT alone, respectively. A KB-xenograft mouse model was used to evaluate the antitumor efficacy of the combined therapy vs. monotherapies: PDT and chemotherapy alone caused 44% and 46% inhibitions of the tumor growth, respectively, while the combined treatment inhibited the tumor growth by 80%. Importantly, therapy with PLNE-PTX nanoemulsions allowed the administration of a 4-fold lower PTX dose (1.8 vs. 7.2 mg/kg of PTX) compared with standard PTX therapy to obtain comparable tumor reduction results. Obviously, the reduced PTX dosage in the PLNE-PTX combination therapy has the potential to reduce the side effects associated to PXT administration.

### 5.3. Combination of PSs with Platinum Compounds

Platinum-containing anticancer agents, with cisplatin, carboplatin, and oxaliplatin as the major and worldwide clinically approved representatives of the category, are largely used alone or in combination for treating various types of cancers. These drugs are very effective but show important side effects, which have stimulated the search of second- and third-generation platinum-based drugs together with strategies optimizing precise delivery to cancer cells also in combination with localized treatment as PDT [138,139,140].

**Scheme 4:**
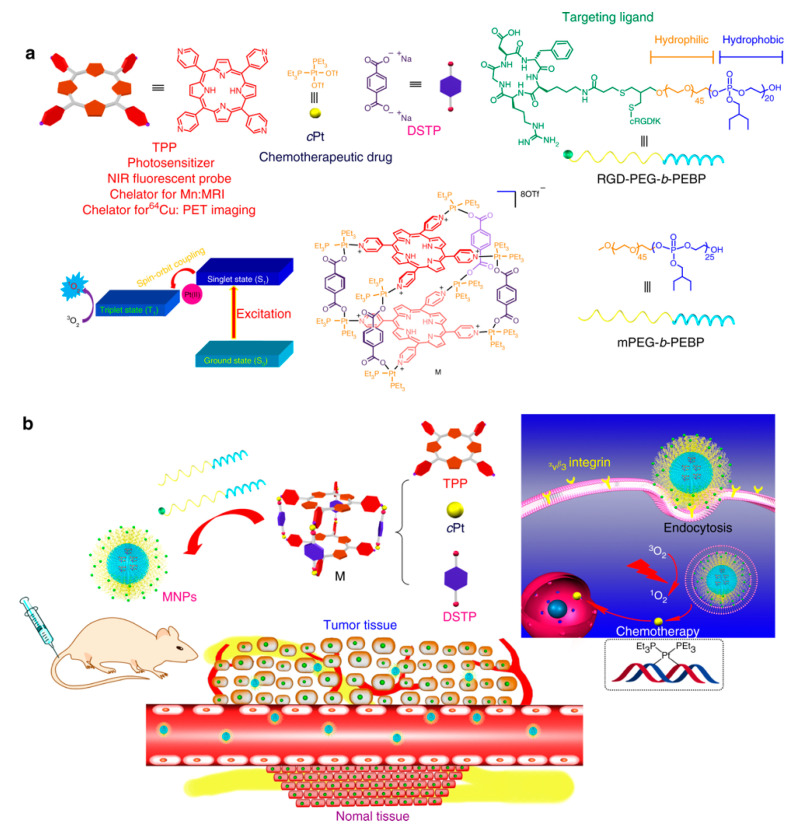
Schematic representation of the mechanisms of action of MNPs developed by Yu et al. [107]. The figure is a reprinted from the original paper. In (**a**) the authors report the chemical structures of the single entities which compose MNPs, while in (**b**) they depict MNPs injection in mice and their accumulation in the tumor mass driven by EPR effect and receptor-mediated endocytosis.

Yu et al. [107] realized a chemotherapy/PDT combination approach using an organoplatinum(II) metallacage (M) formed via a multicomponent coordination-driven self-assembly with 5,10,15,20-tetra(4-pyridyl)porphyrin (TPP), cis-(PEt_3_)_2_Pt(OTf)_2_ (cPt), and disodium terephthalate (DSTP) as the building elements. To improve solubility and stability in physiological conditions and to increase biocompatibility and bioavailability, M was encapsulated into NPs (MNPs) formed by the amphiphilic mPEG-b-PEBP and RGD-PEG-b-PEBP. The MNPs exhibited an increased fluorescence intensity and ^1^O_2_ yield with respect to the free porphyrin, long blood circulation time, and high accumulation in tumors through the EPR effect and active targeting. It was also shown that ^64^Cu and/or Mn ions could be hosted in the TPP-containing cores of MNPs, thus creating a nanoplatform that could be utilized for multiple imaging techniques, such as positron emission tomography (PET) and magnetic resonance imaging (MRI) in addition to near-infrared fluorescence imaging exploiting the porphyrin fluorescence (Scheme 4). In vitro studies on U87MG cells overexpressing αvβ3 integrins showed a higher intracellular accumulation of MNPs with respect to NPs without a cyclic RGD peptide and decreased accumulation of MNPs in cells preincubated with the free peptide to block the αvβ3 receptor; altogether, these observations confirmed receptor-mediated endocytosis and facilitated cellular internalization of MNPs. The cytotoxicity studies demonstrated excellent synergistic effects of chemotherapy and PDT (MNPs plus a 671 nm light) as judged by the combination index CI which was significantly lower than 1 (0.11).

The active targeting properties and the EPR effect of i.v. injected MNPS were demonstrated with in vivo and ex vivo NIR fluorescence imaging studies in U87MG tumor-bearing nude mice. Intense fluorescence signals were observed exclusively in the tumor, whereas they were negligible in lungs, the spleen, and the heart. PET and MRI imaging using ^64^Cu-loaded MNPs and Mn-loaded MNPs, respectively, allowed complementary monitoring of the biodistribution of NPs and confirmed their targeting properties. The pharmacokinetic of MNPs vs. cPt determined by measuring Pt concentration in the blood demonstrated that the circulation half-life of the MNPs was prolonged eight times with respect to that of cPt. Indeed, approximately 3.7% of MNPs dose was still circulating in the plasma 24 h post-injection, while for cPt 98.4% of the administered dose was cleared from bloodstream already 2 h post-injection. As expected, MNPs tumor accumulation was much higher than that of cPt, approaching 1.82 ± 0.17 vs. 0.47 ± 0.06 μg g^−1^ of tissue 24 h post-injection, respectively. The in vivo efficacy studies with a single drug injection demonstrated that only photochemotherapy using MNPs determined the complete eradication of U87MG tumors with no recurrence during the observation period. Moreover, MNPs treatment prolonged the survival of mice over 60 days, while the median survival of mice treated with PBS (control), MNPs (chemotherapy only), and TPP NPs plus light (PDT only) were 22, 38, and 46 days, respectively. Importantly, no body weight loss or other side effects were observed in the mice treated with MNPs and exposed to light, while significant decreases of body weight, nephrotoxicity, pulmonary and hepatic damages were found in mice treated with free cisplatin or cPt. Of note, systemic toxicity of platinum-based drugs can become an important issue in the case of tumors with inherent or acquired resistance, thus requiring high drug doses to obtain efficacy. Importantly, in the above-described paper, Yu and co-workers [107] stressed the fact that photochemotherapy using MNPs could inhibit the growth and ablate completely cisplatin-resistant A2780CIS-derived tumors without inducing any significant systemic toxicity in the treated mice. On the contrary, chemotherapy alone was ineffective, while PDT alone inhibited the growth of A2780CIS tumors, but recurrence was observed during the observation period. In addition, the authors tested whether the combination of chemotherapy and PDT could inhibit/prevent the formation of metastasis of the highly aggressive murine 4T1 breast cancer cells. They showed that slight or moderate anti-metastatic effects were obtained with chemotherapy or PDT alone, while their combination effectively inhibited lung metastasis spread. This efficient inhibition was very likely due to the efficient killing of the cancer stem cells (CSCs) population, highly resistant to chemotherapy and responsible of cancer recurrence and metastasis, as demonstrated in a subsequent study using human hepatocellular carcinoma cells as a tumor model [141]. The authors selected the CD133-positive cancer cell population, possessing CSC-like features, and established CSC-enriched cultures starting from CCLP-1 and Huh7 cell lines. The selected cells were fully characterized to assess CSC properties, such as high efficiency of spheres and tumor formation and expression of transcription factors including OCT4, Sox2, and NANOG, demonstrating the occurrence of synergistic killing when MNPs were used. Besides, the combination therapy using MNPs strongly reduced CSC migration capacity and spheroid formation, according to the observed reduced ability of forming metastasis. Treatments of CSC-derived multicellular spheroids showed that only MNPs plus light induced almost complete killing of the CSCs population, while chemotherapy and PDT alone affected the viability of cells located exclusively in the outer rims of the spheroids. The subcutaneous injection into nude mice of CSCs isolated from in vitro spheroids that received the different treatments showed that the tumorigenic potential was significantly reduced in terms of tumor volume and weight in the case of CSCs derived from spheroids that underwent MNPs-based photo/chemotherapy. Overall, the findings presented in the above-mentioned papers indicated that metallacage-based NPs allows a highly synergic interaction between chemotherapy and PDT, resulting in efficient ablation of primary tumors, including those resistant to cisplatin, eradication of CSCs, and inhibition of metastasis spread. 

To limit the serious side effects of the active Pt(II) drugs, several strategies were attempted including the development of Pt(IV) prodrugs that can be reduced to a Pt(II) cytotoxic compound after reaching the target cells [142]. For prodrugs activation, the selective delivery of light to tumors can be used as an external stimulus, therefore sparing normal tissue from toxic effects of Pt-based compounds [143]. In this context, platinum(IV)-azide complexes appears very promising, because they are stable in the dark and in the presence of glutathione in the millimolar range, while under light irradiation they undergo reduction with the formation of active cytotoxic Pt(II) compounds with concomitant release of dioxygen, which in turn can alleviate hypoxia and reinforce ROS production by PS during PDT [144]. 

**Scheme 5:**
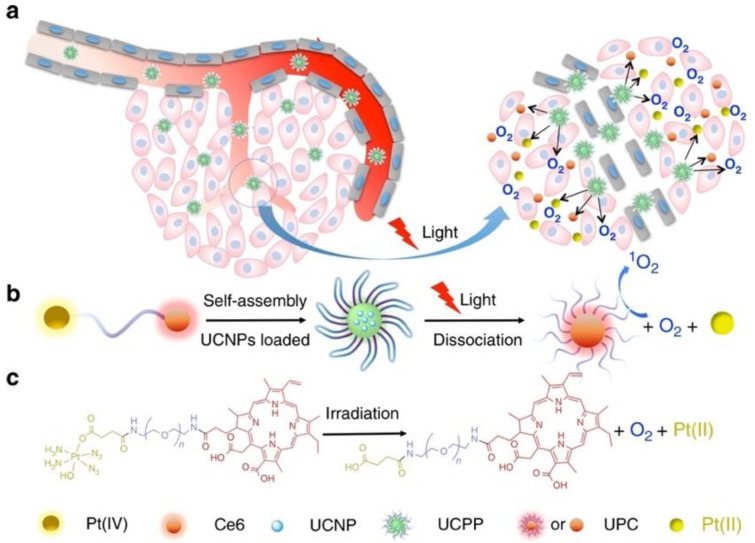
Schematic representation of the mechanisms of action of UCNPs-embedded nanoparticles. The figure is reprinted from Xu et al. [108]. The authors illustrate in (**a**) the light-driven dissociation of UCPP NPs within the tumor micro-environment, in (**b**) UCPP NP synthesis and its light-driven dissociation, which includes O_2_ self-generation, simultaneous activation of PDT, and Pt(II) release for the synergistic photo- and chemotherapy, in (**c**) the mechanism of UCPP NP chemical dissociation.

As an example of this typology of Pt-bases nanosystems combined with the potentiality of upconverting NPs technology, Xu et al. [108] co-assembled the amphiphilic oligomer Ce6-PEG-Pt(IV) (CPP), formed with chlorin Ce6 conjugated via PEG to a Pt(IV) complex, with UCNPs doped with NaYbF4:Tm@CaF2 to obtain UCPP NPs (Scheme 5). 

The authors demonstrated that the 980 nm NIR light absorbed by UCNPs was converted into 365 nm and 660 nm emissions, which efficiently induced the decomposition of Pt(IV) with the concomitant release of O_2_ and the photoactivation of Ce6 with ROS formation, respectively, as illustrated in Scheme 5. Under the 980 nm irradiation, the release of O_2_ could alleviate tumor hypoxia and replace the O_2_ consumed during PDT, providing conditions for realizing strong synergism between PDT and chemotherapy. Indeed, the occurrence of synergism was demonstrated in vitro in HeLa cells incubated with UCPP NPs and irradiated in normoxic and hypoxic conditions; the therapeutic efficacy was significantly higher compared to those of UPC and UPP used as control NPs embedding exclusively PEG-Ce6 and PEG-Pt(IV), respectively. As expected, following i.v. injection, UCPPs exhibited long circulation in the bloodstream and gradually accumulated in the tumor up to 24 h, as shown by in vivo and Ex vivo fluorescence imaging. The anticancer activity of UCPP in vivo was evaluated in two subcutaneous tumor models, derived from HeLa and HCT116 cells, and two superficial carcinomas, derived from B16 and MDA-MB-231 cells. The tumor-bearing mice were intravenously injected with UCPPs or control NPs once every two days for three times, and the tumors were irradiated on day 5. In all cancer models tested, chemotherapy alone using UPP or UCPPs and PDT alone using UPC inhibited the growth of the tumor mass for a few days after treatment, but thereafter a rapid regrowth was observed as the index of slight antitumor efficacy of monotherapies. On the contrary, in mice injected with UCPPs and irradiated with a 980 nm light, tumors completely disappeared, without recurrence within the observation period. Thus, on the basis of these encouraging observations, the authors underlined how the excellent antitumor effect in vivo was achieved by the rational design of UCPPs, which in turn can self-generate O_2_ and Pt(II) under NIR irradiation for relieving tumor hypoxia and favoring synergism of PDT and chemotherapy. Indeed, immunofluorescence analysis of the tumor slices indicated suppression of HIF-1α and CD31 proteins, which is a clear indication of hypoxia reversion. 

As an alternative to Pt prodrugs, in the last years, several authors have proposed combination approaches in which Pt is used in the form of nanoparticles (nano-Pt) serving as a chemotherapeutic agent through the leaching of Pt ions [145], as well as an oxygen-replenishing material based on their catalase-like nanoenzyme property [82,146]. 

Considering the properties of nano-Pt, Liu et al. [83] developed a biomimetic liposomal nanoplatinum formulation for targeted and synergistic chemo- and phototherapy. As reported by the authors, ultrasmall nano-Pt (3–5 nm) were encapsulated in the inner aqueous space of liposomes, while the lipid bilayer was used to load the hydrophobic and clinically used PS verteporfin (VP). The obtained nano-Pt/VP@Lipo was then hybridized with the plasma membranes of RAW264.7 macrophages to obtain biomimetic nano-Pt/VP@MLipo, possessing long circulation features conferred by the CD47 protein and targeting properties of inflamed endothelium conferred by the LFA-1 and Mac-1 leukocyte adhesion proteins [147]. The authors demonstrated that, following i.v. injection, nano-Pt/VP@MLipo accumulated in the tumor where nano-Pt catalyzed the decomposition of H_2_O_2_, providing oxygen enhancing the VP-based PDT effect. In turn, ^1^O_2_ produced during PDT disrupted the liposomal membrane causing the release of nano-Pt, thus improving their penetration into tumor and chemotherapy efficacy. This improved penetration capacity of nano-Pt following irradiation with 690 nm light was demonstrated in 4T1 tumor spheroids and correlated with extensive cell death in the inner core of spheroids. The pharmacokinetic parameters determined by measuring VP plasma concentration in BALB/c mice demonstrated that nano-Pt/VP@MLipo and VP@MLipo had a 1.6-fold longer half-life compared to uncoated nano-Pt/VP@Lipo, highlighting the importance of the biomimetic coating in prolonging circulation time. The ex vivo imaging of excised 4T1 tumors observed 24 h post-injection showed a 2-fold increase of VP fluorescence in animals treated with nano-Pt/VP@MLipo compared to that in those treated with nano-Pt/VP@Lipo. Consequently, VP fluorescence signals in MPS organs exposed to nano-Pt/VP@MLipo were decreased, indicating improved tumor targeting of the biomimetic liposomes favored by the presence of LFA-1 and Mac-1 proteins on NP coating [148]. Tumor vessel targeting was documented by the colocalization of DiI labeled nano-Pt@MLipo with CD31 expressed on the tumor vessels; importantly, this co-localization was not evident with DiI-labeled conventional liposomes. The in vivo antitumor efficacy studies on orthotopic 4T1 breast tumors demonstrated that nano-Pt/VP@MLipo plus irradiation (wavelength: 690 nm; intensity: 100 mW cm^−2^) gave the strongest inhibition of growth of the primary tumor, when compared to other treatments, including PDT alone and chemotherapy alone. The optimal antitumor effects observed using nano-Pt/VP@MLipo were also explained with in situ reversion of hypoxia, as documented by reduced pimonidazole staining and HIF-1α expression in tumor tissues, which would potentially enhance VP-mediated PDT.

### 5.4. Combination of PSs with Topoisomerase Inhibitors (TIs)

Among the TI category and in the field of anticancer nanomedicines, the natural drug camptothecin (CTP) (see Section 4.4) is particularly appealing to be modified and synthetized in the form of prodrugs, often in combination with PSs [149,150,151], and to be further utilized for the formation of carrier-free NPs [152,153]. As an example, Chu and co-workers reported on the synthesis of a novel ROS-responsive nanosystem (MPEG-(TK-CPT)-PPa) derived from the self-assembly of a prodrug of CPT and pyropheophorbide (PPa) both conjugated to a PEG methyl ether (MPEG) via a TK and lipid linkage, respectively [109]. Of note, the nature of the conjugate (e.g., water-insoluble CPT and PPa and highly hydrophilic MPEG) promoted the self-assembly of these NPs in water, with CPT and PPa in the hydrophobic inner core and the MPEG forming the outer shell. The authors systemically investigated the ROS-mediated release of CPT from the prodrug and from MPEG-(TK-CPT)-PPa NPs, highlighting the fundamental role of laser-activated PPa in producing sufficient amounts of ROS able to break the TK linkage. In vitro combination therapy studies performed on HCT116 colon cancer cells revealed a significant synergistic interaction between PPa-PDT and CPT (combination index of 0.14) when MPEG-(TK-CPT)-PPa NPs were used, due to a photodynamic effect combined to the efficient ROS-mediated release of the cytotoxic CPT. Accordingly, HCT116 tumor-bearing mice treated with MPEG-(TK-CPT)-PPa NPs and laser irradiation displayed a significantly higher tumor reduction with respect to mice not exposed to laser. Histological investigations on tumor tissue of MPEG-(TK-CPT)-PPa NPs-treated mice showed an increased number of shrunken cells and pyknosis, leading to high-rate apoptosis and proliferation arrest, while the absence of morphological changes in normal organs or hemorrhage and inflammatory cell infiltration assured the excellent biocompatibility of the prodrug nanosystems proposed by Chu and collaborators. 

Among the synthetic TOPI inhibitors, irinotecan (IRI) is specifically present in the S-phase of the cell cycle [154]. Following administration, IRI is metabolized by esterases and converted to the active metabolite SN-38 [155]. Currently, IRI is used in both its free and liposomal formulations for the treatment of pancreatic and other cancers [156]. Of note, the liposomal formulation improves the circulation half-life, pharmacokinetics, and intratumoral accumulation of IRI and the metabolite SN-38, while minimizing toxic side effects [155]. However, liposomal IRI has been shown to extend only marginally the overall survival of patients with gemcitabine-refractory metastatic pancreatic cancer when combined with fluorouracil and leucovorin [157], thus stimulating investigations on additional combinatorial approaches to treating pancreatic cancers exploiting the full potential of IRI.

In this review, the studies of Hasan’s group [158,159] and that of Anbil [160] highlighted a cooperative mechanistic interaction between IRI-based chemotherapy and low-dose PDT, based on the administration of a PEGylated liposomal formulation of BPD (L-BPD) and a liposomal formulation of IRI (L-IRI) to pancreatic cancer cells in vitro and in vivo. In the above-mentioned combinations of PDT and IRI, the drugs were co-administered but in separated liposomal carriers, exclusively with the rationale of facilitating the translation of the combined approach to the clinic.

However, very recently, Obaid and collaborators [110] reported on the synthesis of a single nanosystem (e.g., a cetuximab antibody-targeted liposome (EGFR-TPMIL)) for the co-delivery of IRI and a lipidated version of BPD for the concomitant pancreatic ductal adenocarcinoma (PDAC) treatment and associated desmoplasia remediation. Indeed, using MIA PaCa-2 cells as an in vitro PDAC model, the authors demonstrated that cetuximab-conjugated TPMIL increased up to 34-folds of the specificity of liposome binding to EGFR-overexpressing cells with respect to the untargeted counterpart (PMIL), thus confirming the tumor specificity of the targeted nanoconstruct as well as the exceptional photo-toxic effect determined after a 690 nm light cells activation (IC_50_ measured for TPMIL decreased by 21-folds with respect to that for untargeted PMIL). It was also demonstrated that light administration is crucial to induce IRI release from TPMIL liposomes, thus increasing the overall cytotoxic effect determined by the synergism between BPD-PDT and IRI-chemotherapy. The following in vivo biodistribution experiments in an orthotopic desmoplastic model of PDAC, obtained by mice inoculation with both MIA PaCa-2 cells and patient-derived pancreatic cancer-associated fibroblasts (PCAFs), demonstrated a 1.5-fold increased tumor-to normal tissue selectivity of TPMIL 12 h post-injection, perivascular penetration up to 105 nm, which was further increased by 11% if the tumor area was exposed to a 690 nm light irradiation. Accordingly, tumor growth inhibition studies revealed that mice which received TPMIL and a 690 nm light (12.5 J/cm^2^ PDT dose) showed almost complete tumor regression, different from those receiving PMIL and the same irradiation scheme, in which the tumor volume increase during observation time was similar to that of untreated mice. Of note, the authors demonstrated that when mice received separated Visudyne plus nal-IRI (e.g., the liposomal FDA-approved BPD and IRI formulation) and a 690 nm light, the tumor growth decrease was 5.6-folds less effective, notwithstanding that the dose of IRI in TPMIL was halved with respect to that present in nal-IRI. Furthermore, in vivo SGH imaging-based desmoplasia remediation studies revealed that only TPMIL and the 690 nm light was able to induce a dramatic tumor collagen density decrease (91%) as well as significant microstructural perturbations, which was finally reflected by the reduction of tumor burden and in doubled and progression-free mice survival. Importantly, this recent study of Obaid and colleagues demonstrated, for the first time, how EGFR targeting combined with the simultaneous co-delivery of IRI and BPD effectively achieved the spatial and temporal synchronization of TOPI inhibition and PDT, allowing PDAC regression by reduction of the tumor mass and concomitant collagen structure perturbation.

Ghosh and collaborators developed a single nanoplatform for the spatiotemporal and light-mediated release of IRI and a porphyrin-phospholipid (PoP) derivative [111]. For the preparation of this unprecedent nanocarrier, the authors considered the lipid composition of ONIVYDE^®^, the liposomal IRI formulation recently approved in United States for treating metastatic pancreatic cancers, as a starting point, and modified it by including pyro-lipid in the bilayer. Interestingly, mice bearing subcutaneous pancreatic MIA PaCa-2 tumors that were injected with one single dose of IRI-PoP liposomes (15 mg/kg IRI) and laser treatment had complete tumor regression and survived without tumor regrowth for 90 days. In contrast, IRI-PoP liposomes administered to mice but not activated with light or IRI-free PoP liposomes activated with laser showed a modest inhibition of tumor progression, and tumor recurrence was measured 65 days post-treatment. Biodistribution and pharmacokinetic studies highlighted that 1 h laser irradiation after IRI-PoP liposomes injection enhanced the delivery of IRI in the tumor by 4-folds with respect to non-irradiated tumors. Importantly, 24 h after IRI-PoP liposomes injection, the level of the active metabolite SN-38 in the tumor of laser treated mice was 7-fold higher with respect to that found in non-irradiated mice (325 ng/g vs. 46 ng/g) as a result of the occurrence of photodynamic-induced vascular damages at the tumor site.

In a similar approach, Turchin’s group proposed a liposomal photoactivatable multifunctional nanoplatform (PMIL) carrying a lipid-anchored derivative of BPD in the bilayer and IRI in an inner aqueous core [112]. In vivo studies on BALB/c mice injected with colon carcinoma CT26 cells demonstrated higher therapeutic efficacy (97% inhibition of tumor growth) following treatment with PMIL compared to BPD containing liposomes (81% inhibition) or IRI containing liposomes (50%). Analogous to the previous example, the authors confirmed that the release of IRI from liposomes was triggered by NIR light and its enhanced accumulation in tumor was mainly mediated by vascular damages. Notwithstanding these encouraging results, it is worth to note that in humans the conversion of IRI to the active metabolite SN-38 was very low (ca. 5%), thus strongly limiting the clinical outcomes. Therefore, several authors reported on the direct NP-mediated delivery of SN-38 [161], often in combination with other drugs.

**Scheme 6:**
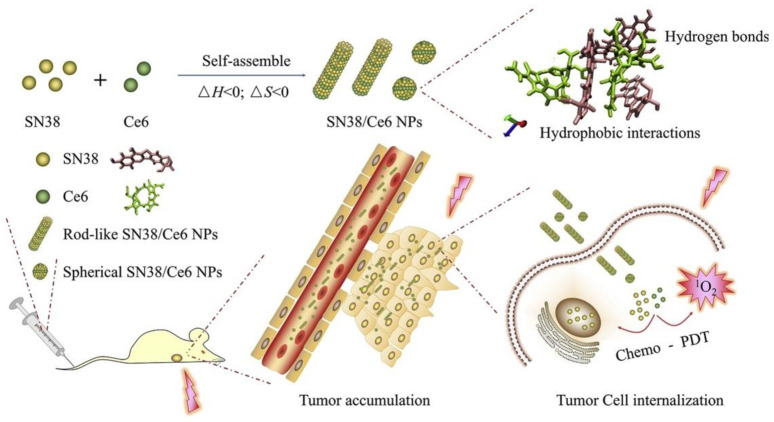
Schematic representation of the mechanisms of action of SN38/Ce6 NPs. The figure is reprinted from Zhao et al. [113].

In this review, Zhao and colleagues used the antisolvent precipitation method to develop carrier-free SN38/Ce6 NPs, exploiting the collaborative assembly of the two insoluble molecules SN38 and Ce6 and obtain NPs with mostly uniform rods in shape, mingled with a few spherical particles (Scheme 6) [113]. SN38/Ce6 NPs and laser irradiation decreased the viability of murine 4T1 breast cancer cells in vitro with significantly higher efficiency in comparison to monotherapies: the calculated CI value of 0.31 indicated a high synergic interaction of Ce6-PDT and SN38. The in vivo efficacy tests were carried out in BALB/c mice bearing subcutaneous 4T1-derived carcinoma and i.v. injected once every two days for 10 days with SN38/Ce6 NPs. PDT alone (Ce6 injection plus laser) and chemotherapy alone (SN38 injection) were poorly effective and produced a tumor inhibition rate of no more than 20%. By contrast, SN38/Ce6 NPs without laser gave an inhibition rate of ∼65 % that was further improved to 85% in the group of mice treated with SN38/Ce6 NPs plus a 660 nm laser. The in vivo biodistribution studies showed that the SN-38 concentration in the tumor was increased by 12-folds after injection of NPs with respect to the metabolite injected in the absence of NPs, and this finding explained the high antitumoral effect of SN38/Ce6 NPs.

As regards our literature survey on NP-mediated TOPII inhibitors delivery in combination with PSs, no examples of compounds other than DOX were judged notable for the scope of the present review.

## 6. Conclusions

In the last two decades, nanotechnological approaches and continuous research on biocompatible materials for drug delivery purposes have completely changed the perspectives of anticancer drug administration. Even if majorly at the pre-clinical stage, the use of precisely designed nanosystems has revealed their huge potential not only in increasing drug bioavailability, but also in reducing side effects and allowing a controlled drug release in the TME, thus counteracting important criticisms of cancer therapy. As reviewed in the present paper, even in the context of chemotherapy and PDT, difficulties in the administration of hydrophobic compounds as well as undesired side effects have been attenuated by drugs inclusion in nanometric delivery systems, which allowed the simultaneous co-transport of chemotherapeutics and PSs to improve tumor delivery, drugs timing, and consequently efficacy. Overall, the examples presented in the review highlighted how an accurate selection of materials endowed with bio-responsiveness or selected for possessing unique prerogatives, sometimes combined with a smart drug design (e.g., TME or ROS-responsive prodrugs) or biomimetic approaches, could effectively maximize tumor reduction in in vivo cancer models with respect to single treatments or with respect to free drug combinations administered in the absence of delivery systems. Nonetheless, notwithstanding the impressive numbers of reports in the field, especially in the last five years, reported in vitro and in vivo preclinical results from completed or ongoing clinical trials foreseeing the use of nanoparticle-based drug formulations still remain restricted to already approved products (e.g., Visudyne^®^ or Doxil^®^ for the liposomal formulation of the PS verteporfin and of DOX, respectively), while no human studies on combination of PDT and chemotherapy are reported. Therefore, an open question remains on the effective limitations that prevent the translation of the proposed approaches from the laboratory scale to clinical trials.

## Figures and Tables

**Table 1:** Summary of the DDSs developed for the co-delivery of PSs and chemotherapeutics selected from the literature reports spanning from 2017 to 2022 and described in detail in the frame of the present review.

Chemo Drug	PS	DDS Composition	DDS Features	Cancer Model	Outcome	Reference
Doxorubicin (DOX)	IR780	Self-assembled micelles composed of a conjugate (oxygen carrier (F), IR780, and hydrophilic PEG chain) loaded with DOX	Hypoxia relief	MCF7 human breast cancer	Enhanced in vivo tumor regression in mice treated with O_2_-loaded micelles	[95]
Doxorubicin (DOX)	Tetraphenylporphyrin (TPP)	Polymeric nanoparticles (PDPA-TPP-DOX)	pH responsiveness	4T1 murine breast cancer	Improved uptake and retention in an in vivo tumor site (lower pH)	[96]
Doxorubicin (DOX)	Pheophorbide A (PhA)	Self-assembled prodrug nanoparticles (PEG-TK-DOX) loaded with PhA	ROS responsiveness	CT26 murine colon cancer	Higher accumulation of DOX in an in vivo tumor site	[97]
Doxorubicin (DOX)	Chlorin e6 (Ce6)	Self-assembled prodrug nanoparticles (CRGDK-PEG-DOX) loaded with Ce6	pH responsiveness, active targeting through neuropilin-1 (NRP-1) receptor-mediated internalization	MDA-MB-231 and MCF7 human breast cancer	Increased uptake in MDA-MB-231 (NRP-1^+^), compared to MCF7 (NRP-1^−^), and longer in vivo blood circulation and intratumoral accumulation	[98]
Doxorubicin (DOX)	Chlorin e6 (Ce6)	Polymeric nanoparticles (PEI-NI and HA-Ce6) loaded with DOX	Targeting of CD44^+^ cells and hypoxia responsiveness	LLC murine lung carcinoma	Recognition of CD44^+^ cells and consequent endocytosis, leading to higher uptake of targeted NPs, and strong in vivo anticancer effect in hypoxia	[99]
Doxorubicin (DOX)	Rose Bengal (RB)	Biomimetic upconverting nanoparticles (PEG-TK-DOX and RB co-loaded upconverting NP core, coated with cancer cell membranes)	ROS responsiveness and tumor infiltration	4T1 murine breast cancer	Preferential in vitro accumulation in homologous cancer cells and immunogenic cell death induction and stronger in vivo tumor growth inhibition	[100]
Paclitaxel (PTX)	Chlorin e6 (Ce6)	Human serum albumin (HSA) nanoparticles loaded with catalase (Cat), PTX, and Ce6	Hypoxia relief	4T1 murine breast cancer	Prolonged blood circulation (stealth properties due to HSA), increased in vivo intratumoral oxyhemoglobin levels, and low expression of hypoxia markers	[101]
Paclitaxel (PTX)	Pheophorbide A (PheoA)	Carrier-free self-assembled prodrug (PTX_2_S) micelles loaded with PheoA	Redox and ROS responsiveness	MDA-MB-231 human breast cancer and SKOV-3 human ovarian cancer	Micelles disassembly in simulated reductive TME	[102]
Paclitaxel (PTX)	Pheophorbide A (PheoA)	Carrier-free self-assembled prodrug (PTX_2_S/PTX-SS-PEG-MAL) micelles loaded with PheoA	Redox and ROS responsiveness and HSA-mediated uptake	MDA-MB-231, MCF7 human breast cancer, and 4T1 murine breast cancer	Higher in vitro intracellular uptake of HSA-binding micelles	[103]
Paclitaxel (PTX)	Tetraphenylchlorin (TPC)	Biomimetic polymeric (PEG-*b*-PDLLA) nanoparticles loaded with PTX prodrug (PTX_2_-TK) and TPC, coated with red blood cell (RBC) membranes	Redox responsiveness and stealth properties	HeLa human cervix adenocarcinoma and RAW264.7 murine macrophages	Reduced macrophage capture of RBC-coated NPs, longer in vivo blood circulation, and reduced hepatic clearance	[104]
Docetaxel (DTX)	Chlorin e6 (Ce6)	Self-assembled nanoparticles (core composed of modified hyaluronic acid and Ce6) loaded with DTX	Targeting of CD44^+^ cells and redox responsiveness	MCF7 human breast cancer (2D cell cultures) and 4T1 murine breast cancer (tumor-bearing mice)	NPs disassembly in reductive environment, higher uptake of Ce6 NPs in MCF7 CD44^+^ cells, and persistent intratumoral retention in vivo	[105]
Paclitaxel (PTX)	Porphyrin-lipid	Porphyrin-lipid nanoemulsion loaded with PTX, stabilized DSPE-PEG2000	Stealth properties	KB human epithelial carcinoma	Prolonged blood circulation and enhanced intratumoral retention	[106]
*cis*-Platin (cPt)	Tetrapyridylporphyrin (TPP)	Self-assembled coordination complex between cPt, TPP, and DSTP loaded in NPs composed of mPEG-b-PEBP and RGD-PEG-b-PEBP	Active targeting through ανβ3 receptor-mediated uptake	U87MG human glioblastoma	In vitro and in vivo studies on U87MG cells overexpressing αvβ3 integrins showed a higher intracellular accumulation of MNPs	[107]
Platinum (IV) complex	Chlorin e6 (Ce6)	Upconverting nanoparticles loaded with Ce6-PEG-Pt (IV) conjugate	Hypoxia relief	HeLa human cervix adenocarcinoma (2D cell cultures and xenografted mice), HCT116 human colorectal cancer, B16 murine melanoma, and MDA-MB-231 human breast cancer	In vivo suppression of HIF-1α and CD31 hypoxia markers	[108]
Nano-platinum (nanoPt)	Verteporfin (VP)	Biomimetic liposomes (lipid bilayer containing VP) loaded with nanoPt and coated with RAW264.7 macrophage membranes	Hypoxia relief and tumor infiltration	4T1 murine breast cancer (2D and 3D cell cultures; tumor-bearing mice)	Efficient tumor site targeting, with increased efficacy of VP-PDT due to hypoxia reversion by nanoPt catalase-like feature	[83]
Camptothecin (CPT)	Pheophorbide A (PPa)	Self-assembled nanoparticles (composed of a conjugate MPEG-(TK-CPT)-PPa)	ROS responsiveness	HCT116 human colorectal cancer	Reduced premature drug release and delivery of the two drugs to the tumor site, where their release was enhanced by ROS production following irradiation	[109]
Irinotecan (IRI)	Lipidated benzoporphyrin derivative (BPD-PC)	Liposomes with lipidated BPD in the bilayer, functionalized with cetuximab and loaded with IRI	Active targeting of EGFR^+^ cells	MIA PaCa-2 human pancreatic cancer, i.e., MIA PaCa-2 + PCAF (xenografted mice)	Improved efficacy in EGFR^+^ tumors and desmoplasia remediation	[110]
Irinotecan (IRI)	Porphyrin-phospholipid derivative (PoP)	Liposomes with PoP bilayer loaded with IRI	Light-controlled release of the chemotherapeutic	MiaPaCa2 human pancreatic cancer (xenografted mice)	Light exposure resulted in increased intratumoral level of active metabolite SN-38	[111]
Irinotecan (IRI)	Lipidated benzoporphyrin derivative (BPD)	Liposomes with lipidated BPD in the bilayer and loaded with IRI	Light-controlled release of the chemotherapeutic	CT26 murine colorectal cancer (tumor-bearing mice)	Light exposure resulted in increased intratumoral level of active metabolite SN-38	[112]
Ethyl-10-hydroxycamptothecin (SN-38)	Chlorin e6 (Ce6)	Antisolvent-precipitated nanoparticles	Increase drug solubility	4T1 murine breast cancer (2D cell cultures; tumor-bearing mice)	High drug loading, excellent stability in aqueous solutions, and enhanced intracellular and intratumor uptake and retention	[113]
